# Mrs. Mary C. Miner

**Published:** 1903-04

**Authors:** 


Mrs. Mary C. Miner, widow of Dr. Julius F. Miner, died at her home in Buffalo, February 23, 1903, aged 77 years. The death of Mrs. Miner removes one of the last remaining personages of the earlier period of Buffalo University memory, in which, as the consort of Professor Miner, she played an important part. She was held in great esteem by the older alumni, who mourn her loss and revere her memory.

